# Serum 25-Hydroxyvitamin D Is Inversely Associated with Monocyte Percentage to HDL Cholesterol Ratio among Young Healthy Adults in Qatar

**DOI:** 10.3390/nu13010127

**Published:** 2020-12-31

**Authors:** Hanaa Mousa, Nazmul Islam, Vijay Ganji, Susu M. Zughaier

**Affiliations:** 1 College of Medicine, QU Health, Qatar University, Doha P.O. Box 2731, Qatar; hanaa.mousa@qu.edu.qa; 2 #2Biomedical and Pharmaceutical research Unit, QU Health, Qatar University, Doha P.O. Box 2731, Qatar; 3 Public Health Department, College of Health Sciences, QU Health, Qatar University, Doha P.O. Box 2731, Qatar; nislam@qu.edu.qa; 4 Human Nutrition Department, College of Health Sciences, QU Health, Qatar University, Doha P.O. Box 2731, Qatar; vganji@qu.edu.qa

**Keywords:** vitamin D, 25-hydroxyvitamin D, HDL, monocyte percentage, MHR, inflammation

## Abstract

Low serum 25-hydroxyvitamin D [25(OH)D] is linked to an altered lipid profile. Monocytes play an important role in inflammation and lipid metabolism. Recently, monocyte percentage to HDL-cholesterol ratio (MHR) has emerged as a novel marker of inflammation. We investigated the association between serum 25(OH)D concentrations and MHR and serum lipids in young healthy adults. Data from the Qatar Biobank were utilized to investigate the relation between serum 25(OH)D and inflammation and serum lipid concentrations in healthy Qatari adults using multivariate regression analysis. Prevalence of serum 25(OH)D concentrations <12 ng/mL (deficiency), 12–20 ng/mL (insufficiency), and ≥20 ng/mL (sufficiency) were 55.8%, 29.9%, and 14.3%, respectively. Serum 25(OH)D was significantly inversely associated with monocyte percentage, MHR, total cholesterol, LDL-cholesterol, and triacylglycerol in multivariable adjusted analysis. MHR could be a potential biomarker to predict cardiometabolic diseases among young healthy Qataris.

## 1. Introduction

The classical function of vitamin D is to maintain the homeostasis of calcium and phosphorous. Vitamin D can be obtained from the diet and skin exposure to the sun’s UVB light. Regardless of the source, in the liver, vitamin D is converted to 25-hydroxyvitamin D [25(OH)D], a major circulatory vitamer. Further, in the kidney, 25(OH)D is converted to 1,25 dihydroxyvitamin D (1,25(OH)_2_D) by 25-hydroxyvitamin D-1α-hydroxylase [1]. Non-calcemic effect of vitamin D is through the action of 25(OH)_2_D via vitamin D receptors. Low circulating concentrations of 25(OH)D is highly prevalent in Qatar. Previous studies conducted in 2012 reported an estimated 90% of the Qatari population suffer from various degrees of vitamin D insufficiency and deficiency [2]. More recently, Al-Dabhani reported that 64% of the population in Qatar suffers from vitamin D deficiency [3].

The impact of low vitamin D on health outcomes is well documented [4]. Specifically, vitamin D deficiency is related to several chronic inflammatory diseases [5] such as metabolic syndrome, obesity, and cardiovascular diseases (CVD) [6,7]. Therefore, by improving serum vitamin D may lead to reduced inflammation and CVD [8]. Zughaier et al. [9] reported that the hormonally active 1,25(OH)_2_D leads to a significant decrease in IL-6 and IL-1β gene expression in monocytes exposed to an inflammatory stimulus such as lipopolysaccharide.

Monocytes are central immune cells that display a wide range of homeostatic and immune response functions. Because monocytes are major secretors of the pro-inflammatory mediators such as cytokines, an increase in monocytes percentage may be an indication of sub-clinical inflammation [10,11]. Additionally, HDL is a major component of total cholesterol that has been known to have anti-inflammatory and protective functions, hence dubbed as the good cholesterol [12]. Thus, when monocyte percentage is elevated and HDL is reduced, monocyte to HDL cholesterol ratio (MHR) is elevated which suggests a homeostatic perturbations and sub-clinical inflammation. Additionally, vitamin D deficiency has been linked to dyslipidemia such as low HDL-cholesterol [13,14,15]. However, it is not very clear about the mechanism through which vitamin D exerts its effects on lipids [16,17].

Recently, MHR has been recognized as a novel biomarker of subclinical inflammation [18,19,20]. The total number of monocytes is inversely related to HDL and MHR is reported to be elevated in many disorders such as hypertension [21], atherosclerosis [22], and diabetic nephropathy [23]. Elevated MHR is associated with disease severity in CVD such as coronary artery stenosis [24,25]. MHR is also associated with a chronic inflammatory condition such as chronic obstructive pulmonary disease [26]. MHR was reported in many studies as an independent prognostic biomarker in many diseases associated with high inflammatory status particularly disorders linked to cardiac disorders or atherosclerotic events. For example, in infective endocarditis patients, MHR alone predicted the in-hospital death with relatively higher sensitivity (74.4%) and specificity (57.6%) [27]. In acute ischemic stroke, patients with higher MHR showed higher susceptibility to develop clinical complications such as intracranial hemorrhage [28]. However, the association between serum 25(OH)D and MHR is not known yet. Therefore in this study, we investigated the relationship between serum 25(OH)D concentration and subclinical inflammation biomarker, MHR in healthy young adults in Qatar.

## 2. Materials and Methods

### 2.1. Study Design and Study Participants

This study is a cross-sectional, retrospective study based on the data collected by the Qatar Biobank (QBB). The study sample broadly represents the population of Qatar. Briefly, the QBB collected data on Qataris and non-Qataris who have been living in the country ≥15 years. The participants were ≥18 years old. Data on general health and lifestyle, diet, cognitive function, and physical and clinical measurements were collected. Further, several health and clinical biomarkers were measured in blood, urine, and saliva. The detailed methodologies were described elsewhere [29,30]. Ethical IRB approval (QBB-RES-ACC-0237-0142) and confidentiality agreements were obtained before conducting this study. The total number of participants in this study was 874; however 14 participants were excluded as gender was missing. Hence, the initial analysis included 860 participants (men, *n* = 399; women, *n* = 461).

The inclusion criteria were young healthy Qatari adults between the ages of 18 and 40 years who did not have any co-morbidities. The exclusion criteria included those using vitamin D supplements and those with CVD, diabetes, hypertension, kidney disease, liver disease, pregnancy, cancer, and critical illness. Thus, the sample contained apparently healthy subjects. Vitamin D status was defined as deficiency, insufficiency, and sufficiency if the serum 25(OH)D concentrations were <12 ng/mL, 12-˂20 ng/mL, ≥20 ng/mL, respectively. This classification was based on the Institute of Medicine’s recommendations [31].

Physical and biochemical measurements: Participants’ height, weight, and BMI were measured with light clothing by trained nurses. Bodyweight was measured using the TANITA BC-418 MA instrument. BMI was computed using weight in kg divided by height in m^2^. Venous blood samples were collected from participants after overnight fasting. Blood specimens were sent to Hamad Medical Corporation Laboratories (College of American Pathologist Accredited Laboratory) for further analysis. Monocytes, white blood cells (WBC), lymphocytes, and neutrophils were measured as part of the differential while blood cell count. All the blood biomarkers such as serum 25(OH)D, plasma glucose, serum HDL cholesterol, serum total cholesterol, serum LDL cholesterol, and serum triacylglycerol were measured all at once. Serum 25(OH)D concentration (included both vitamin D_2_ and vitamin D_3_ fractions) was measured using electrochemiluminescence immunoassay (LIAISON^®^ 25-hydroxyvitamin D Total Assay, DiaSorin Inc., Stillwater, MN, USA). Plasma glucose was measured with the enzymatic/amperometric method (Nova statstrip and Roche Accucheck Inform II devices). Serum total cholesterol was measured with enzymatic CHOD-PAP method. HDL cholesterol Plus Third Generation Method was used to measure the serum HDL cholesterol. LDL cholesterol Plus Second Generation Method was used to measure the serum LDL cholesterol. Serum triacylglycerol was measured with enzymatic GPO-PAP method. Detailed methodologies were described elsewhere [29,32].

### 2.2. Statistical Analysis

Baseline characteristics according to the serum 25(OH)D concentrations were presented as frequencies and percentages for categorical variables and as means (SD) for continuous variables. Data were tested for normality using Shapiro–Wilk test. Histograms were constructed to detect the normality. Although MHR and 25(OH)D concentrations were not normally distributed based on the significance, the histogram revealed that the data were very close to normal. Therefore, regression analysis was performed on non-transformed data. Comparisons between participants with serum 25(OH)D concentrations (sufficient, insufficient, and deficient) were performed using chi-squared Tests (or Fisher exact tests for cells <5) for categorical variables. ANOVA was used for normally distributed numerical variables and Kruskal–Wallis tests were used for non-normally distributed interval variables. Accordingly, Chi-Squared was used for gender, while ANOVA was used for age, BMI, WBC, monocyte, lymphocyte, neutrophil, neutrophil percentage to HDL ratio (NHR), total cholesterol, LDL, and HDL. Kruskal–Wallis test was used for MHR, lymphocyte percentage to HDL ratio (LHR), WBC percentage to HDL ratio, and triglycerides. Additionally, baseline characteristics between men and women were also reported for selected characteristics. The differences between men and women for monocyte percentage, serum HDL cholesterol, and MHR were tested with an independent, 2-tailed *t*-test.

An association between serum25(OH)D and inflammatory markers and serum lipids were analyzed with multinomial logistic regression using gender, age, BMI, and smoking as confounding variables. In this analysis, the sample size varied from 702 to 706 depending on the variable. We also performed a forward step-wise multinomial regression adding each confounding variable at a time to study the impact of each confounding variable on the relationship between serum 25(OH)D and MHR. In the multinomial logistic regression analysis, 25(OH)D ≥ 20 ng/mL category was used as a reference category. Multinomial logistic regression coefficients (β) and their 95% confidence intervals (95% CI) were reported. Further, an association between serum 25(OH)D concentrations and inflammatory markers and serum lipids were analyzed with multivariable linear regression using gender, age, BMI, and smoking as confounding variables. In this analysis, all serum 25(OHD, inflammatory markers, and serum lipids were used as continuous variables. Multivariable regression coefficients (β) and their 95% confidence intervals (95% CI) were reported. Additionally, we performed a restrictive Cubic Spline adjusted regression analysis to assess whether the relationship between serum 25(OH)D concentrations and MHR was linear. For simplicity, we did not report cubic spline analysis between serum 25(OH)D and other inflammatory markers and serum lipids. All statistical analyses were two-sided. A *p* ˂ 0.05 was considered statistically significant. Analyses were performed using the Stata statistical software package 16 (Stata Corp, College Station, TX, USA).

## 3. Results

### 3.1. Serum Vitamin D Concentrations in Young Healthy Adults in Qatar

Table 1 shows the baseline characteristics based on serum 25(OH)D concentrations. In this study, the prevalence of serum 25(OH)D <12 ng/mL and ≥20 ng/mL were 55.8% (*n* = 488) and 14.2% (*n* = 125), respectively. The mean BMI was significantly higher in the vitamin D deficient group (28.3 kg/m^2^) in comparison with a sufficient group (26.4 kg/m^2^). Monocyte percentage was significantly higher in participants with vitamin D deficiency compared to sufficiency. In the vitamin D sufficient group, the MHR mean was signifcantly lower (5.1) compared to the vitamin D deficiency group (5.8) or the vitamin D insufficiency group (5.7) (*p* ˂ 0.011).

**Figure 1:**
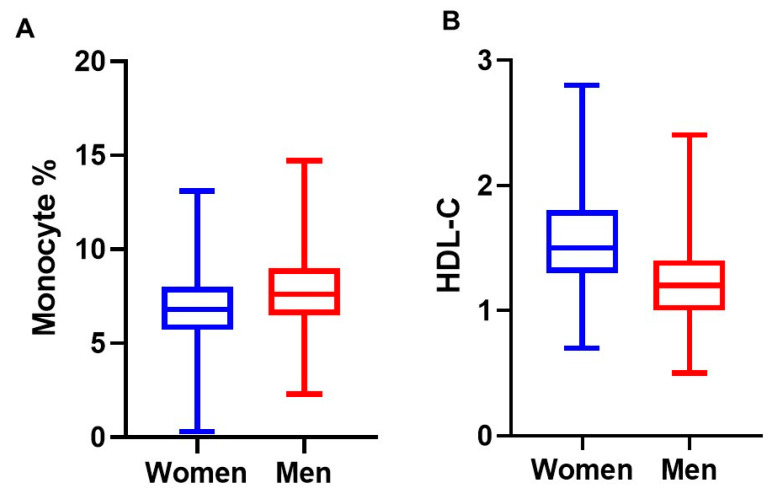
Differences in selected baseline characteristics between men and women. (**A**): Monocyte percentages in women and men (6.95 vs. 7.87; *p* < 0.001 for independent, 2-tailed *t*-statistic). (**B**)**:** HDL cholesterol concentrations in women and men (1.56 vs. 1.24 mmol/L; *p* ˂ 0.001 for independent, 2-tailed t-statistic).

**Figure 2:**
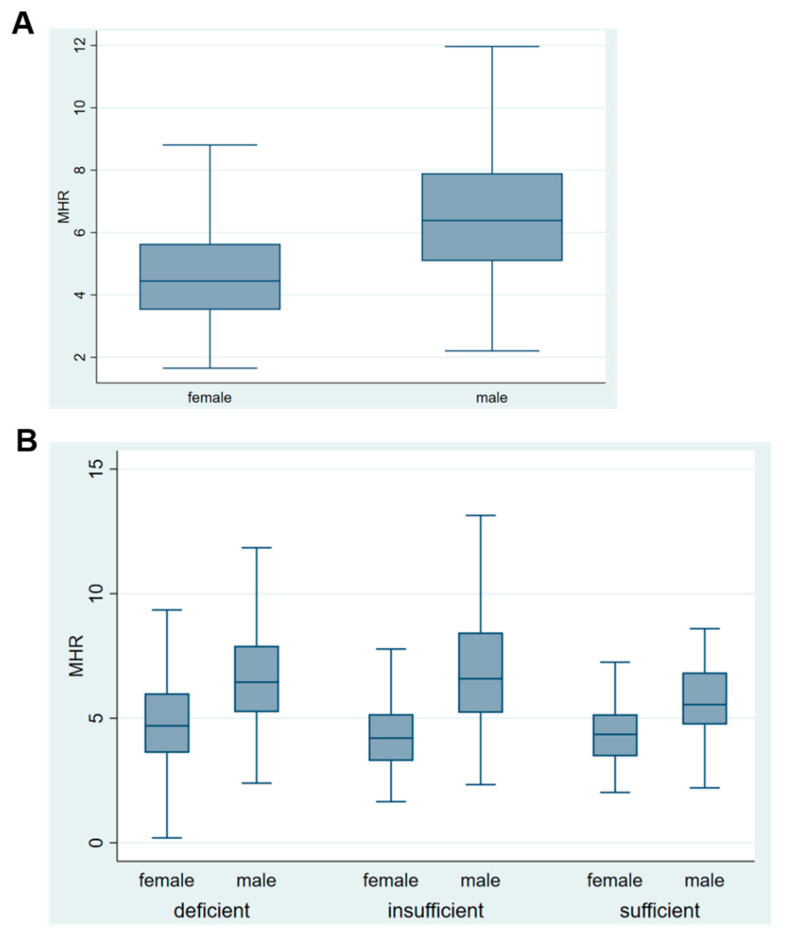
Comparison of MHR based on serum 25(OH)D concentrations in healthy young adults in Qatar. Serum 25(OH)D concenrtations were defined as deficiency (<12 ng/mL), insufficiency (12-˂20 ng/mL), and sufficiency (≥20 ng/mL). (**A**): Boxplots of MHR in women compared to men (*p* ˂ 0.001 for *t*-statistic). (**B**): Boxplots of MHR for men and women within vitamin D deficient (*p* ˂ 0.004 for independent, 2-tailed *t*-statistic), vitamin D insufficient (*p* ˂ 0.58 for independent, 2-tailed *t*-statistic), vitamin D sufficient (*p* ˂ 0.21 for independent, 2-tailed *t*-statistic) categories. To convert ng/mL to nmol/L, multiply with 2.496. Abbreviations: 25(OH)D, 25-hydroxyvitamin D; MHR, monocyte percentage to HDL cholesteorl ratio; VitD, vitamin D.

Additionally, gender differences in selected characteristics were described in Figure 1 and Figure 2. Monocyte percentage was significantly lower in women compared to men, while the HDL was significantly higher in women compared to men (*p* ˂ 0.001). The MHR was significantly lower in women compared to men (Figure 2A). However, the MHR was significantly lower in women compared to men in vitamin D deficient group (*p* ˂ 0.004) but not in vitamin D insufficiency or in sufficiency group (Figure 2B).

### 3.2. Association between Serum 25(OH)D Concentrations and Inflammation Biomarkers and Serum Lipids

The associtioan between serum 25(OH)D concentrations and inflammatory markers and serum lipids (categorized form) are presented in Table 2. In the multivariabe regression analysis, a significant association between serum 25(OH)D and MHR was observed in the deficient group, (β = 0.19; *p* ˂ 0.005) in comparison to the sufficient category. Moreover, in vitamin D insufficient participants, the regression coefficient was 0.15 (*p* ˂ 0.03). The monocyte percentage coefficient was statistically significant in vitamin D deficient participants 0.19 (*p* ˂ 0.006) in comparison to the vitamin D sufficient category. In contrast, no associations were observed between serum 25(OH)D concentration and lymphocyte percentage, neutrophil percentage, LHR, and NHR. Interestingly, in vitamin D deficient participants, serum lipids such as serum total cholesterol (*p* ˂ 0.014), serum LDL-cholesterol (*p* ˂ 0.03), and serum triacylglycerol (*p* ˂ 0.04) but not serum HDL-cholesterol were significantly associated with serum 25(OH)D. Plasma glucose was not significantly assocaited with serum 25(OH)D concentrations.

Additionally, the multivariable associations between serum25(OH)D and inflammatory biomarkers and serum lipids (continuous form) are presented in Table 2. We observed a significant inverse relation between serum 25(OH)D and monocytes percentage, (β = −0.54; *p* ˂ 0.002), MHR, (β = −0.38; *p* ˂ 0.008), serum total cholesterol, (β = −1.03; *p* ˂ 0.009), serum LDL cholesterol, (β = −0.98; *p* ˂ 0.026), and serum triacylglycerol (β= −1.06; *p* ˂ 0.028). The relationship in continuous variable regression analysis, between serum 25(OH)D and monocytes percentage, MHR, and serum total cholesterol, LDL cholesterol, and triacylglycerol was much stronger compared to the multinomial logistic regression.

**Figure 3:**
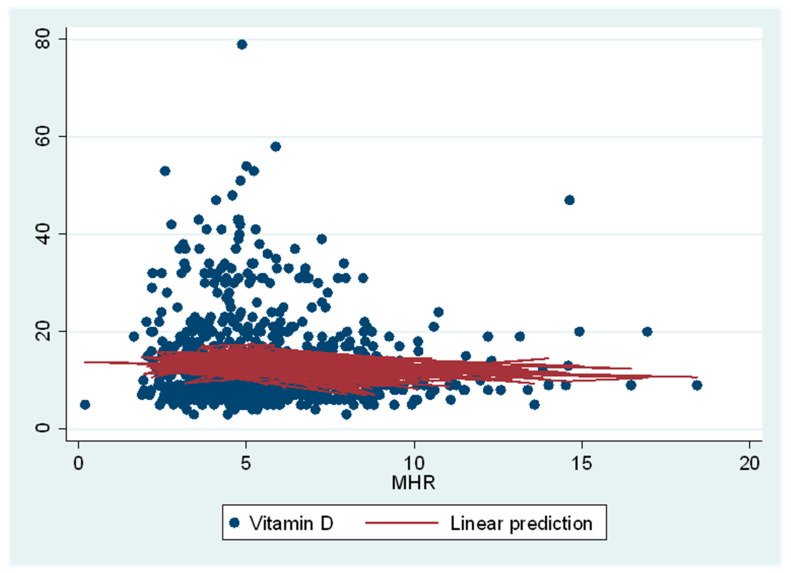
Restricted Cubic Spline Regression between serum 25(OH)D concentration and MHR after adjustment for age, gender, BMI, and smoking in young health adults in Qatar (n = 701). Serum 25(OH)D and MHR were used as continuous variables. Linear relationship between serum 25(OH)D and MHR is significant (*p* ˂ 0.001). Abbreviations: 25(OH)D, 25-hydroxyvitamin D; MHR, monocyte percentage to HDL cholesterol ratio.

Serum 25(OH)D had a linear relationship with MHR in Restricted Cubic Spline multivariable adjusted regression analysis when analyzed as continuous variables. In both logarithmic transformed and non-transformed analyses, we observed a significant linear relationship between serum 25(OH)D and MHR (*p* ˂ 0.001) (Figure 3). In stepwise forward regression analyses, age, gender, BMI, and smoking were significantly related in all regression models. However for simplicity, in Figure 3, we only reported the multivariable adjusted relationship between non-transformed serum 25(OHD and MHR in a continuous form (without categorization of serum 25(OH)D concentrations).

## 4. Discussion

We have investigated the relationship between serum 25(OH)D concentrations and MHR and serum lipids in healthy young Qatar population. This is the first study to report an inverse relationship between 25(OH)D and MHR. This relationship was measured in using 2 separate statistical procedures, i.e., stepwise multinomial adjusted logistic regression using serum 25(OHD as categorical variable and multivariable adjusted linear regression using 25(OH)D and inflammatory markers and serum lipids as a continuous variables. In categorized analysis, vitamin D deficiency was significantly related to MHR. In the continuous regression, serum 25(OH)D was significantly, inversely related to MHR and serum lipids. It is interesting to note that based on regression coefficients, the relationship was much stronger in the continuous regression analysis.

MHR is an emerging novel maker of CVD [33] such as ischemic stroke [34] and cerebral hemorrhage [35]. Elevated MHR is an indicator of systemic inflammation as well as oxidative stress [18]. In disorders like polycystic ovary syndrome, MHR was found to be more sensitive than the usual markers such as increased BMI and C-reactive protein (CRP) in predicting disease development [36]. Moreover, MHR is reported to predict the severity and complication in diseases such as obstructive sleep apnea, and MHR was used to anticipate the cardiovascular sequels [37]. MHR also demonstrated efficiency in predicting short-term mortality in patients with ST-segment elevation myocardial infarction [38].

Elevation in monocyte percentage can occur in infection, inflammation, and other cellular perturbations such as autoimmunity disorders, thus reflecting clinical inflammation [39]. Monocyte percentage elevation suggests more perturbation and inflammatory cytokine release, whereas absolute monocyte number vary among participants and gender. Therefore, in this study, monocyte percentage is used to better reflect sub-clinical inflammation status. Vitamin D is known to exert anti-inflammatory effects on monocytes leading to reduced proinflammatory cytokines release and reprogramming of cells [9,40]. Vitamin D deficiency is observed in various chronic inflammatory diseases indicating the anti-inflammatory effect of vitamin D [41] We found a significant inverse association between serum vitamin D and the subclinical inflammation marker, MHR, among healthy young adults. This relation could be explained by several mechanisms. Vitamin D is a suppressor of endoplasmic reticulum stress leading to downregulation of adhesion molecules such as PSGL-1, β(1)-integrin, and β(2)-integrin, consequently, decreasing the monocytes activation [42]. Furthermore, vitamin D possesses an immunomodulatory effect and regulates monocyte inflammatory responses by attenuating cellular signaling and pro-inflammatory genes activation, subsequently preventing cytokines release. For example, Vitamin D attenuates TLR2 and TLR4 mediated signaling leading to reduction in TNFα release, and activation of intracellular inflammatory pathways like p38 and NF-kB pathway [43].

Epidemiological studies showed a significant relation between low concentrations of serum 25(OH)D and the risk of infections and hospitalization [44,45]. For instance, the risk of acute lower respiratory tract infection is reported to be higher in children who suffer from vitamin D deficiency in the first two years of life [46]. In support, vitamin D supplementation demonstrated a protective effect against acute respiratory tract infections [47]. Further, vitamin D deficiency is prevalent in several inflammatory diseases, such as inflammatory bowel disease [48], and rheumatoid arthritis [49]. In systemic lupus erythematosus, vitamin D induces reconstruction of the balance between B and T cells through stimulating an increase in CD4+ T cells and a decrease of memory B cells and anti-DNA antibodies [50]. Moreover, in cancer patients, a meta-analysis study illustrates an enhanced overall survival in patients who have higher serum 25(OH)D concentrations [51]. The given data suggest that the importance of vitamin D as an anti-inflammatory and an antioxidant nutrient metabolite. A recent retrospective study found a negative relation between serum 25(OH)D concentrations and CRP (a marker of inflammation and cytokine storm), in COVID-19 patients, which again indicates a protective role of vitamin D in reducing inflammation [52].

The association between vitamin D deficiency and dyslipidemia, i.e., low HDL, high LDL, and high triacylglycerol is well documented [15]. Studies reported a significant association between serum 25(OH)D concentrations and HDL. Subjects with sufficient serum 25(OH)D concentrations have higher concentrations of HDL, while deficient subjects have significantly lower HDL concentration [13,53]. Further, sufficient HDL is shown to be protective in CVD and other chronic diseases [54]. However, in this young healthy adult cohort, we did not observe any significant associations between serum 25(OH)D concentrations and the HDL profile as this sample is selected to be devoid of co-morbidities, which may explain the lack of association in this study. Additionally, it has been known that anti-dyslipidemia drugs affect HDL cholesterol concentrations. Consequently, MHR measurements would be affected accordingly. However, we were unable to investigate the potential confounding effect on the relationship between serum 25(OH)D and MHR as this study sample did not contain subjects who were taking anti-lipidemic medications. Further, this might be interesting to study this confounding effect in older cohort.

The importance of this study is that it highlights the association between serum 25(OH)D concentrations and the novel biomarker MHR. This association could be utilized to predict the risk of progressing into diseases like metabolic syndrome and dyslipidemia. Recent studies investigated whether lymphocytes and neutrophils as predictors of inflammation. LHR and NHR along with MHR were good predictive biomarkers of inflammatory diseases such as metabolic syndrome [55,56]. In our study, we found no association between serum 25(OH)D and LHR and NHR. This is probably due to sample selection criteria; young healthy adults who do not have any underlying disorders. However, thus far, no study reported an association between 25(OHD concentrations and LHR and NHR.

The cross-sectional design of the study is one of the study limitations. Therefore, the cause and effect should not be assumed. Moreover, this study is conducted on healthy young adults, which explains the modest changes in HDL and MHR. If the study population is older, we may have a higher MHR and perhaps a stronger association between serum 25(OH)D concentrations and MHR. Further, in this study, we did not correct for general confounders like socioeconomic status as the cohort are all Qataris without drastic differences in demography, and with relatively high socioeconomic status. Therefore, the lack of adjustment for this variable, might not affected the outcome. Future studies are warranted to confirm the association of serum vitamin D with MHR in an older population with or without chronic inflammatory conditions. In conclusion, serum 25(OH)D concentrations is inversely associated with the MHR, a novel subclinical inflammation biomarker.

## Figures and Tables

**Table 1:** Characteristics of the study population based on serum 25(OH)D concentrations (*n* = 860) ^1.^

	Vitamin D Deficiency (serum 25(OH)D, <12 ng/mL)	Vitamin D Insufficiency (serum 25(OH)D, 12-˂20 ng/mL)	Vitamin D Sufficiency (serum 25(OH)D, ≥20ng/mL)	*p*-Value ^2^
N	488	261	125	
Gender ^3^				
Women	274 (56%)	131 (51%)	56 (46%)	ns
Men	214 (44%)	130 (50%)	55 (44%)	
Age (years)	28.8 (6)	30.3 (5.9)	29.8 (5.8)	0.002
Body mass index, kg/m^2^	28.3 (6.8)	27.6 (5.3)	26.4 (5.5)	0.008
White blood cells, cells/10^9^ L	6.8 (2.0)	6.8 (2.0)	6.7 (1.8)	ns
Monocyte, %	7.5 (1.9)	7.3 (1.9)	6.9 (1.6)	0.014
Lymphocyte, %	35.6 (8.6)	35.1 (9.4)	36 (9.1)	ns
Neurtophil, %	53 (9.8)	54.1 (10.4)	53.5 (10.1)	ns
Monocyte % to HDL ratio	5.8 (2.3)	5.7 (2.5)	5.1 (1.8)	0.011
Lymphocyte % to HDL ratio	27.3 (10.1)	26.9 (11.1)	26 (8.4)	ns
Neutrophil % to HDL ratio	41 (14)	42 (16.2)	39 (12.2)	ns
Total cholesterol, mmol/L	4.8 (0.8)	4.8 (0.7)	4.6 (0.8)	ns
HDL-cholesterol, mmol/L	1.4 (0.4)	1.4 (0.4)	1.4 (0.3)	ns
LDL- cholesterol, mmol/L	2.8 (0.8)	2.8 (0.7)	2.7 (0.7)	ns
Triacylglycerol, mmol/L	1.2 (0.7)	1.1 (0.7)	1.0 (0.5)	ns
Glucose, mmol/L	5.0 (0.7)	5.0 (0.9)	4.9 (0.7)	ns

^1^ Data are presented in mean (±SD) for continuous measures and n (%) for categorical measures; ns: not significant. To convert ng/mL to nmol/L, multiply with 2.496. ^2^ Serum vitamin D categorization was based on Institute of Medicine guidelines. ^3^ Significance in Chi-Squared test for proportions or ANOVA for continuous measurements.

**Table 2:** Association between serum 25(OH)D concentrations and inflammatory markers and serum lipids in young healthy adults in Qatar ^1^.

	Vitamin D Deficiency (˂12 ng/mL) ^2^		Vitamin D Insufficiency (12-˂20 ng/mL) ^2^		Continuous Association ^3^	
	β (95% CI)	*p*-Value	β (95% CI)	*p*-Value	β (95% CI)	*p*-Value
White blood cell, cells/10^9^ L (*n* = 702)	0.01 (−0.11, 0.14)	ns	0.02 (−0.11, 0.15)	ns	0.09 (−0.22, 0.41)	ns
Monocyte % (*n* = 702)	0.2 (0.06, 0.34)	0.006	0.09 (−0.05, 0.24)	ns	−0.54 (−0.19, −0.2)	0.002
Lymphocyte % (*n* = 702)	−0.01 (−0.03, 0.02)	ns	−0.02 (−0.04, 0.01)	ns	0.01 (−0.06, 0.08)	ns
Neurtophil % (*n* = 702)	−0.01 (−0.03, 0.02)	ns	0.01 (−0.01, 0.04)	ns	0.02 (−0.03, 0.08)	ns
Monocyte % to HDL ratio (*n* = 701)	0.19 (0.06, 0.32)	0.005	0.15 (0.02, 0.29)	0.03	−0.38 (−0.66, −0.1)	0.008
Lymphocyte % to HDL ratio (*n* = 701)	0.01 (−0.02, 0.04)	ns	0.01 (−0.02, 0.04)	ns	−0.02 (−0.08, 0.04)	ns
Neutrophil % to HDL ratio (*n* = 701)	0.02 (−0.004, 0.04)	ns	0.01 (−0.01, 0.02)	ns	0.001 (−0.04, 0.04)	ns
Total cholesterol, mmol/L (*n* = 706)	04 (0.08, 0.72)	0.014	0.25 (−0.09, 0.58)	ns	−1.03, −1.81, −0.26)	0.009
HDL-cholesterol, mmol/L (*n* = 706)	−0.2 (−0.94, 0.54)	ns	−0.22 (−0.1, 0.58)	ns	0.54 (−1.42, 2.4)	ns
LDL- cholesterol, mmol/L (*n* = 703)	0.4 (0.05, 0.74)	0.03	0.27 (−0.1, 0.63)	ns	−0.98 (−1.84, −0.12)	0.026
Triacylglycerol, mmol/L (*n* = 706)	0.49 (0.02, 0.93)	0.04	0.31 (−0.16, 0.78)	ns	−1.06 (−2.0, −0.11)	0.028
Glucose, mmol/L (*n* = 706)	0.2 ( 0.18, 0.58)	ns	0.1 (−0.26,0.55)	ns	−0.34 (−1.2, 0.49)	ns

^1^ Persons with chronic diseases and who were taking prescription medication were not included in the study. To convert ng/mL to nmol/L, multiply with 2.496. Abbreviations: 25(OH)D, 25-hydroxyvitamin D; β, regression coefficient; ns, not significant. ^2^ Multinomial logistic regression analysis was adjusted for gender, age, BMI, and smoking. Serum Vitamin D categorization (deficiency, insufficiency, and sufficiency) was based on Institute of Medicine guidelines. Vitamin D sufficiency (≥20 ng/mL) was used as a referent category. ^3^ Multivariable regression analysis was adjusted for gender, age, BMI, and smoking. Serum 25(OH)D and inflammatory markers and serum lipids were used as continuous variables.

## Data Availability

Restrictions apply to the availability of these data. Data was obtained from Qatar Biobank (https://www.qatarbiobank.org.qa) under confidentiality agreement with Qatar University.
